# Asymmetric Mach–Zehnder Interferometer Based Biosensors for Aflatoxin M1 Detection

**DOI:** 10.3390/bios6010001

**Published:** 2016-01-06

**Authors:** Tatevik Chalyan, Romain Guider, Laura Pasquardini, Manuela Zanetti, Floris Falke, Erik Schreuder, Rene G. Heideman, Cecilia Pederzolli, Lorenzo Pavesi

**Affiliations:** 1 Nanoscience Laboratory, Department of Physics, University of Trento, Via Sommarive 14, 38123 Povo (TN), Italy; romain.guider@unitn.it (R.G.); lorenzo.pavesi@unitn.it (L.P.); 2 LaBSSAH, Fondazione Bruno Kessler, Via Sommarive 18, 38123 Povo, Italy; pasqua@fbk.eu (L.P.); mzanetti@fbk.eu (M.Z.); pederzo@fbk.eu (C.P.); 3 LioniX B.V., Enschede 7522, The Netherlands; f.h.falke@lionixbv.nl (F.F.); f.schreuder@lionixbv.nl (E.S.); r.g.heideman@lionixbv.nl (R.G.H.)

**Keywords:** Mach–Zehnder interferometer, biosensor, Aflatoxin M1, limit of detection, Fab′

## Abstract

In this work, we present a study of Aflatoxin M1 detection by photonic biosensors based on Si_3_N_4_ Asymmetric Mach–Zehnder Interferometer (aMZI) functionalized with antibodies fragments (Fab′). We measured a best volumetric sensitivity of 10^4^ rad/RIU, leading to a Limit of Detection below 5 × 10^−7^ RIU. On sensors functionalized with Fab′, we performed specific and non-specific sensing measurements at various toxin concentrations. Reproducibility of the measurements and re-usability of the sensor were also investigated.

## 1. Introduction

In recent years, integrated optical sensors have shown very promising results for label-free detection [1]. Even if Surface Plasmon Resonance (SPR) is the most commonly used technique for label-free analysis [2], devices like ring resonators [3,4] and Mach–Zehnder interferometers [5] are showing high sensitivities and miniaturization abilities, which allow realizing a complete lab-on-chip device [6].

We are here interested in a biosensor for a fast and comprehensive detection of Aflatoxin M1 (AFM1) in milk. In fact, the AFM1 mycotoxin is a milk contaminant and a potent carcinogen substance that is regulated by the European Commission (EC No. 1881/2006). At present, the level of AFM1 in milk is analyzed using screening tests like Enzyme-Linked ImmunoSorbent Assay (ELISA). In addition, High-Performance Liquid Chromatography is needed when samples show suspect concentration of AFM1. The combination of these analyses is expensive, time-consuming and bulky.

The sensor, which we are here proposing, is based on an asymmetric Mach–Zehnder Interferometer (aMZI), with silicon nitride (Si_3_N_4_) as core material and SiO_2_ as cladding material. They are fabricated by standard CMOS processing and can be assembled with fluidics in a compact package. The basic idea behind the sensor is that the interference at the output of an aMZI is affected by the phase difference between the light which travels along the two interferometer arms. By opening a window in the SiO_2_ and exposing one of the two arms to an analyte, the change in the refractive index in the window region is measured as an interference fringe shift in the aMZI output signal. From the fringe shift, the phase shift suffered by the light that travels in the exposed arm can be determined. From the phase shift, the change of the refractive index is measured, *i.e.*, the sensor can sense the analyte. The Mach–Zehnder interferometer is made asymmetric by the addition of a small path length difference between both arms. This difference in path length, results in a wavelength dependency of the output phase. Scanning the aMZI over a small bandwidth results in a phase plot of which the phase shift, due to a refractive index change, can be monitored with high accuracy. 

One key point of a biosensor is its specificity. In order to develop a biosensor specific to the detection of AFM1, a functionalization process based on antigen-binding fragments (Fab′) was applied on the aMZI surface. One benefit of using antibodies is the extreme specificity associated with their antigen binding sites [7]. The immobilization of antibodies includes whole antibodies, F(ab)_2_, and Fab′ fragments. Antibodies need to be immobilized in a controlled and oriented fashion, in order to maximize the number of available antigen-binding sites and prevent surface degradation. Although significant progress has been made with whole antibody immobilization, methods for the immobilization of Fab′ fragments have become relevant especially for biosensor development [8,9,10]. Fab′ fragment immobilization offers several benefits over whole antibodies. Firstly, bound Fab′ fragments are able to maintain a higher amount of binding sites per unit area compared to whole antibodies due to their smaller size. Moreover, Fab′ fragments are immobilized easily in an oriented conformation via the nucleophilic sulfide near their C-terminals. The characteristics of Fab′ fragments make them ideal candidates for biosensing, also because the currently well-developed knowledge of the available immobilization chemistries for many different materials that are frequently used as biosensing platforms (e.g., gold, Si, Si alloys, plastic, and inorganic substrates).

In this work, we measured and analyzed the selectivity and regeneration of functionalized asymmetric Mach–Zehnder interferometric biosensors with purified solutions. First, we setup the functionalization procedure of the sensor. Then, we performed sensing measurements using two mycotoxins, Aflatoxin M1 and Ochratoxin, in buffer solution for various concentrations, and, finally, we performed regeneration measurements using glycine-methanol solutions. Our results are confirmed by experiments on functionalized Si_3_N_4_ flat substrates.

## 2. Experimental Section

### 2.1. Materials

3-mercaptopropyltrimethoxysilane (MPTMS, 99%) purchased from Gelest Ltd. (Maidstone, Kent, UK), was used without any further purification. Toluene anhydrous (99.8%), toluene, dithiothreitol (DTT) and all powders for buffered solutions were purchased from Sigma-Aldrich s.r.l. (Milan, Italy). Methoxypolyethyleneglycolthiol (mPEG-SH) with 2000 and 5000 molecular weight were purchased from Nektar Therapeutics AL (Huntsville, AL, USA). A rabbit polyclonal anti-AFM1 antibody and an horseradish peroxidase (HRP)-conjugated Aflatoxin M1 (AFM1-HRP) contained in the I’screen Afla M1 milk Elisa kit were purchased from Tecna s.r.l. (Padua, Italy), while Aflatoxin M1 and Ochratoxin were purchased from Sigma-Aldrich s.r.l. (Milan, Italy). SuperSignal West Femto Chemiluminescent Substrate was purchased from Thermo Scientific (Rockford, IL, USA).

### 2.2. Functionalization Process

F(ab′)_2_ fragments were generated by protease digestion (Immobilized Papain Thermo Scientific) of 20 μL of 1 mg/mL anti-AFM1 polyclonal rabbit IgG according to manufacturer’s instructions. Then, a 13.3 μM F(ab′)_2_ solution was mixed with 10 mM DTT to reduce the disulfide bond in the hinge region, and the mixture was incubated for two hours at room temperature. The mixture was poured into a centrifugal filter unit (Microcon YM-10, MWCO 10000, Millipore Corp., Billerica, MA, USA) to remove the excess of DTT.

The Fab′ were immobilized on the Si_3_N_4_ surface adapting the protocol described in Yoshimoto *et al*., [11] with some modifications. In order to introduce thiol groups able to react with the cysteine groups on Fab′ fragments, the silicon nitride surface was functionalized in wet conditions with mercaptosilane (MPTMS) [12]. The surfaces (both aMZI chip and flat samples) were cleaned with an argon plasma (6.8 W, one min) to remove organic contaminants and to hydroxylate the surface and were then immersed in a 1% v/v solution of MPTMS in toluene anhydrous at 60 °C for 10 min. Silane-coated substrates were rinsed several times with toluene and then dried in a stream of nitrogen. The immobilization of Fab′ fragments onto the surface was carried out by deposition of 80 μL of a 0.33 μM Fab′ in 10 mM phosphate buffer with 10 mM ethylenediaminetetraacetic acid (EDTA). After two minutes, the surface was PEGylated by addition first of 200 μM final concentration of mPEG-SH 5000 (for 30 min on orbital shaker at 80 rpm) and then of 200 μM of mPEG-SH 2000 (for 60 min on shaker at 80 rpm). The surface was finally cleaned using a PBS-EDTA buffer. The concentration of the Fab′ was determined by measuring their absorbance with a Nanodrop instrument, (Extinction coefficient (0.1%) = 1.35 at 280 nm).

### 2.3. Mach–Zehnder Fabrication Process and Design

The aMZI devices are based on the TriPlex technology [13]. A 4” inch 525 μm thick silicon substrate is oxidized until 8 micron thermal oxide layer is formed on the surface. Then, a single 103 nm thick LPCVD (low-pressure chemical vapor deposition) Si_3_N_4_ layer (refractive index 2.02) is deposited onto the thermal oxide followed by a thin LPCVD SiO_2_ layer. This layer stack is patterned by using photolithography, dry etching (RIE) and subsequently resist removal. The waveguides are then covered with a thick 6 μm LPCVD SiO_2_ cladding. To enable an interaction between the evanescent field of the light propagating through the waveguide and the liquid sample of interest, the top cladding is locally removed by opening the sensing window. This is accomplished by a photolithography step and BHF wet etch down to the Si_3_N_4_ layer [14].

**Figure 1:**
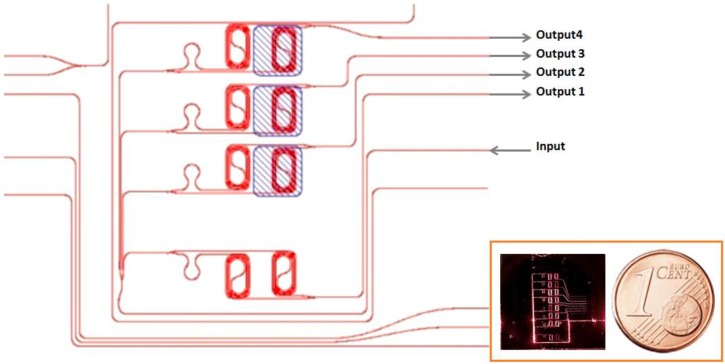
Sketch of the asymmetric Mach–Zehnder interferometer chips measured; (**inset**) photograph of the chip and a one euro-cent coin for comparison. The sensor here is an eight aMZI sensor.

The design of the sensor is reported in Figure 1. In this design, four aMZI are integrated in a single chip. A same input signal is sent to the four aMZI by a one to four channel splitter. Other designs have up to eight aMZI integrated in the same chip (see the inset to Figure 1). The long optical path length of the sensing arms is 6.25 mm, and it is achieved by a spiral waveguide to minimize required surface area. There is a small addition in the path length in the reference arm to obtain the asymmetry. The additional length determines the free spectral range (FSR) of the aMZI and is chosen such that it matched with the bandwidth of the used Vertical-Cavity Surface-Emitting Laser (VCSEL). Openings are shown in the figure by a blue hatched region. Three out of the four aMZI have the sensing window on top of the sensing arm. The fourth aMZI is left covered by the cladding in order to isolate it from the microfluidic chamber and, therefore, to be used as the reference sensor (baseline sensor). This aMZI is used as an internal reference both for the input signal intensity as well as for setting the temperature reference. The area of and pitch between the sensors are chosen such that each individual sensor can be functionalized by a spotter with a different chemistry allowing internal consistency tests or multianalyte detection. Note that the input and output waveguides are all on the same edge of the chip.

### 2.4. Experimental Setup

To characterize the sensors, we used a fiber array to waveguide alignment setup where a 12 fiber array unit with 250 micron pitch between the fibers is placed on multiaxis piezo-controlled translation stages. The light signal polarization is controlled by a two-paddle polarization controller. An optical microscope coupled to a visible/IR camera is used for alignment and imaging. For the detection, we connected the fibers to Si transimpedance amplified photodetectors interfaced to a PicoScope 4824 (an 8 channel USB oscilloscope). Finally, a ULM850-B2-PL VCSELs from Philips Technologies GmbH U-L-M Photonics connected to a single mode visible fiber is used as light source. Wavelength variation is achieved by current tuning the VCSEL with a periodic current ramp which also triggers the time scan of the oscilloscope. In this way, a wavelength scan of the four aMZI output waveguides is recorded by the oscilloscope and transferred to the control computer. The data are analyzed online by an automatic routine to extract the time dependent phase shift signal of each four aMZI. At the end, a live recording of the phase shift of the signal light propagating in the four aMZI is achieved with a VCSEL modulation frequency of 20 Hz, and a data acquisition of 50,000 points per spectrum. For sensitivity and sensing measurements, the chip is sealed on a homemade polydimethylsiloxane (PDMS) microfluidic flow cell with a volume chamber of less than 0.5 µL connected to a VICI M6 liquid handling pump.

## 3. Results and Discussion

### 3.1. Optimization of the Functionalization Process

**Figure 2:**
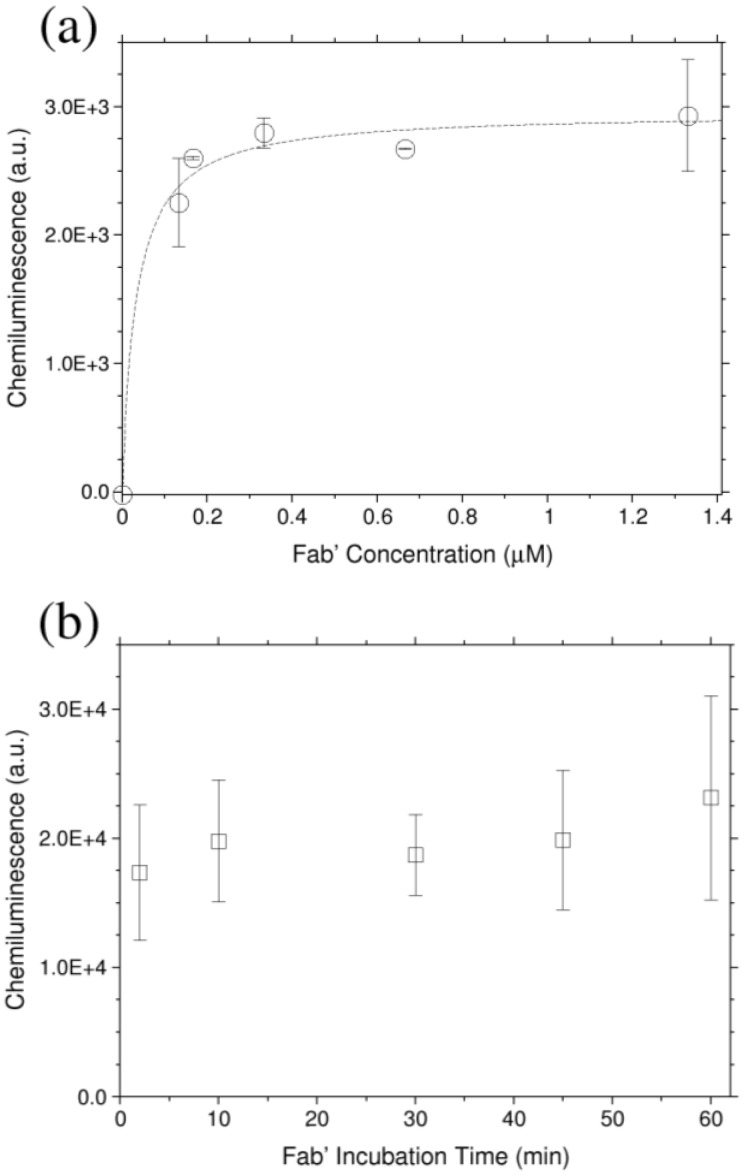
(**a**) Chemiluminescence detection of AFM1-HRP incubated on Fab′ prepared surface. The protocol described in Section 2.2 was applied changing the Fab′ concentration (incubation time 2 min). (**b**) For a 0.33 μM Fab′ concentration, the AFM1-HRP recognition was evaluated as a function of the delay before PEG addition (incubation time). The reported values represent the mean value on two different experiments, and error bars are reported as the standard deviation. Exposition times are equal to 0.2 s and 1 s for (a) and (b), respectively.

The Fab′ density on the silanized Si_3_N_4_ flat surfaces was optimized incubating different concentrations using the protocol reported in Section 2.2. After immobilization, the surfaces were incubated with an HRP-conjugated Aflatoxin M1 (AFM1-HRP) stock solution diluted 80 times in 50 mM MES buffer pH 6.6 for one hour, washed twice in buffer and transferred to a black microplate, where the developer solution was added. HRP in presence of a suitable substrate develops a chemiluminescence signal that can be easily detected. After five minutes incubation, the signal was recorded with a ChemiDoc MP system (Biorad). Figure 2a shows the chemiluminescence signal generated by AFM1-HRP immobilized on functionalized surfaces as a function of the Fab′ concentration. The immobilization protocol described in Section 2.2 was applied, incubating the Fab′ solution for 2 min before PEG addition. No signal is recorded on the PEG passivated surface (without Fab′ solution, data not shown). Fitting the data with a Langmuir equation that describes the adsorption of molecules on a surface, it is possible to determine the surface saturation. A 0.33 μM Fab′ concentration was selected for the following experiments.

In order to optimize the functionalization process, Fab′ are immobilized changing the incubation time from 2 to 60 min before PEG addition. The delayed addition of PEG after Fab′ immobilization is crucial in gold surface functionalization since it prevents a wrong Fab′ orientation [11] while on mercaptosilanized surfaces, we have demonstrated that this is not true. As reported in Figure 2b, in fact, no significant variation in the AFM1-HRP detection is observed, suggesting that 2 min of delay in the PEG addition is enough to obtain a good Fab′ orientation, although no significant effect in the antigen recognition is observed.

### 3.2. Sensing Measurements

**Figure 3:**
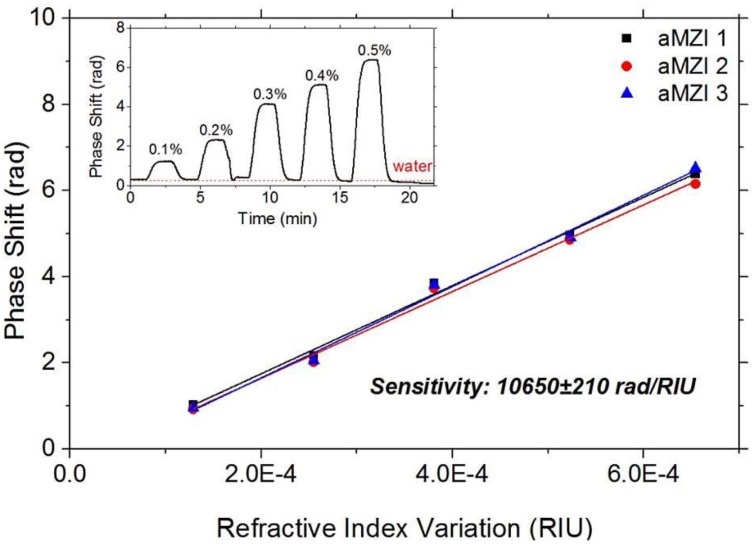
Volume sensitivity measurements. Evaluation of the bulk sensitivity; (**inset**) phase shift curve for one of the aMZI sensors during the injection of the water-glucose solutions (glucose concentration in %w/w labelled on the plot).

In order to define the performances of our photonic sensors, we characterized the volume Sensitivity (S) of the uncovered aMZI. To calculate this parameter, we monitored in real-time the phase shift of the aMZI, as one arm of the sensor was exposed to glucose-water solutions of various concentrations. Similar experiments were already performed by our group on ring resonators, and more details on this experiment are available in [15]. Figure 3 represents the phase shifts as a function of the bulk refractive index variations measured simultaneously on three aMZI. We note that adding the glucose-water solution causes a significant phase shift which is similar for the three sensors on the chip. The initial phase is recovered when only water is flown. From the relation between the glucose concentration (*i.e*., refractive index of the cladding liquid) and the phase shift, we deduced the sensitivity. We found sensitivity of 10,600 rad/RIU with an error of 3%. If we define the limit of detection (LOD) as the minimum input quantity that can be distinguished with more than 99% fidelity, then LOD = 3σ/S, where σ is the output uncertainty, given as the measured phase standard deviation obtained on repeated measurements of blank solution (when no analyte is present). In our sensor, LOD = 5 × 10^−7^ RIU. These values for sensitivity and LOD show good performances of our sensors in comparison with other MZI platforms [16,17].

### 3.3. Aflatoxin M1 Measurements

To perform sensing measurements on these sensors, we initially filled the microfluidic chamber with a buffer solution. In our case, the buffer solution was 50 mM MES buffer pH 6.6. We then injected 75 µL of a solution containing the targeted mycotoxin (AFM1 or Ochratoxin, which is another mycotoxin of similar molecular weight) at a known concentration in order to measure the evolution of the phase of the aMZI due to the capture of the toxin from the functionalized aMZI. Note that the functionalization aims at detecting AFM1 and not Ochratoxin, so this experiment allows testing the specificity of the sensor response. The solution is inserted into the microfluidic chamber using an injection loop in order to avoid the formation of air bubbles in the microfluidic chamber during the buffer exchange. All measurements were done with a flow rate of 3 µL/min.

**Figure 4:**
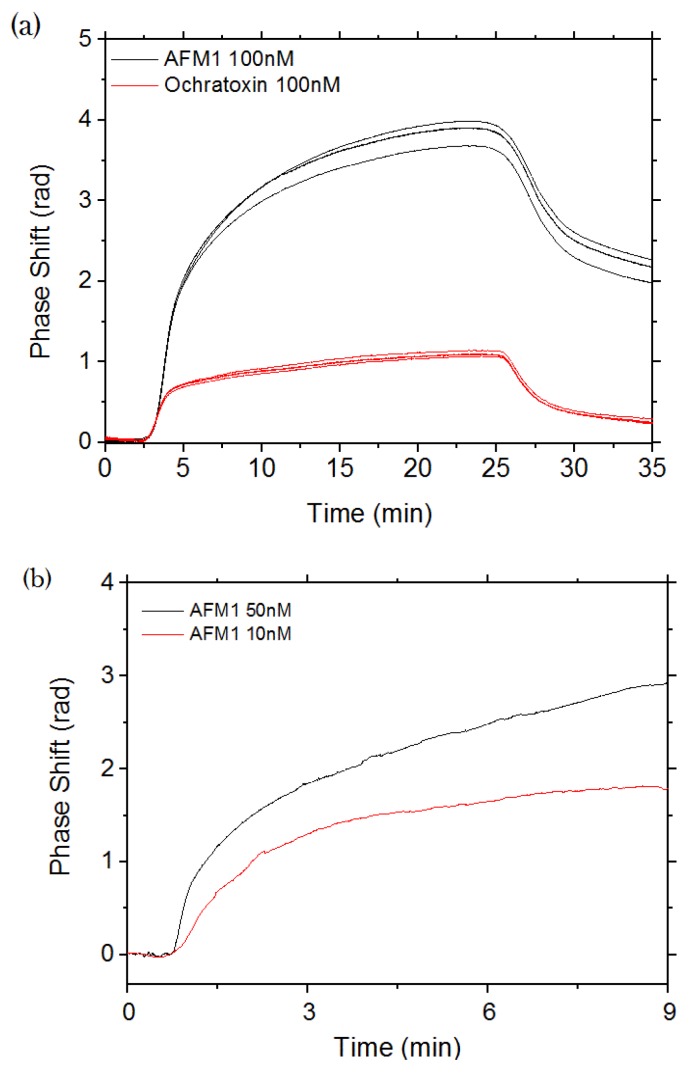
(**a**) Sensorgram recorded on aMZI sensors by flowing AFM1 (black) and Ochratoxin (red) through the microfluidic chamber. At t = 0, MES buffer is flowing through the sensors. The toxin was then injected at *t* = 2.5 min and at *t* = 25 min. The toxin flow is stopped and the MES buffer was injected again; (**b**) Sensorgram recorded on one aMZI sensor by flowing a 50 nM (black) and 10 nM (red) AFM1 solutions in the microfluidic chamber. The phase shift is proportional to the Aflatoxin M1 concentration. The actual phase shift is different from the one in (a) since a different sensor was used.

As is the case of sensitivity measurements, we monitored the phase shift due to the binding of the toxins to the surface. Figure 4a shows the results for the three sensors when a 100 nM concentration of AFM1 (black lines) or of Ochratoxin (red lines) is added to the buffer solution. The phase shifts in time following the kinetics of the binding of the toxins to the antibody on the surface of the exposed aMZI arms. When the toxin flow is stopped, the phase decreases according to the dissociation kinetics of the toxin from the antibodies. These measurements were done on the same chip after one regeneration cycle for the AFM1 and two for Ochratoxin, in order to allow their comparison (see Section 3.4).

We can appreciate the clear dependence of these signals as a function of the injected toxin concentration. The functionalization is shown to be specific. In fact, in the case of AFM1, after MES rinsing, the phase shift is 2 radians larger than the one before the toxin injection, while in the case of Ochratoxin, it is only about 0.25 radians.

In order to determine the lowest concentration of Aflatoxin M1 that could be detected, we performed measurements with different concentrations of Aflatoxin M1. Figure 4b shows the results for 50 nM and 10 nM Aflatoxin M1 concentrations. Considering the molecular weight of Aflatoxin M1 of 328.27 g/mol, 10 nM corresponds to 3 ng/mL. This value is higher than the value permitted by European regulations (50 ng/L in milk). In order to decrease the limit of detection of the sensor, a pre-concentration module is needed. Combining the sensor sensitivity and specificity with a pre-concentration module, the required levels could be accomplished.

### 3.4. Regeneration Measurements

**Figure 5:**
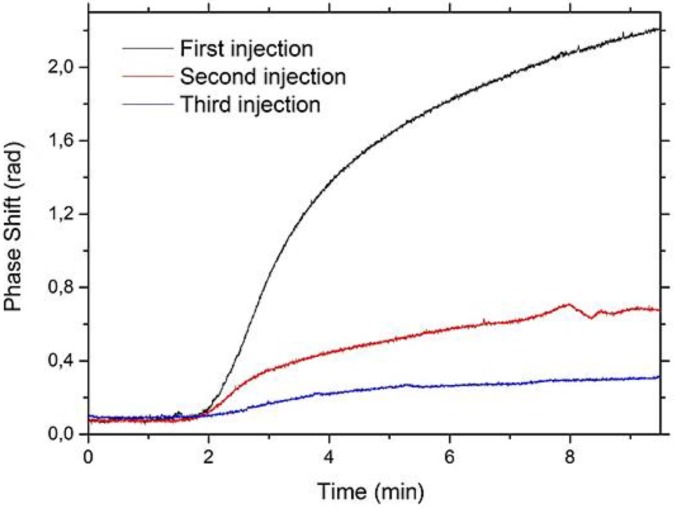
Sensorgram recorded on one single aMZI sensor by flowing a 100 nM AFM1 solution in the microfluidic chamber. The first curve is the response of the fresh sensor, (**black line**), the second one after one glycine –methanol injection (**red line**) and the third one after two injections (**blue line**).

In order to test the reusability of the biosensor, we also investigated the regeneration of the functionalized samples by using 100 mM glycine-HCl pH 2.3 with 10% v/v of methanol (glycine-methanol solution). We injected Aflatoxin M1 solutions in the microfluidic chamber, in order to link the toxin to the functionalized surface of the sensor. We then injected the MES buffer again in the microfluidic chamber for several minutes, in order to restore a stable signal, and finally we injected glycine-methanol solution, in order to break the Aflatoxin-antibody bond and remove all the linked toxins from the sensor surface while keeping the antibodies in place: *i.e.*, we aim to regenerate the sensor. We repeated this procedure several times on the same chip, and analyzed the sensor response. Results are reported in Figure 5 for a 100 nM Aflatoxin M1 concentration.

**Figure 6:**
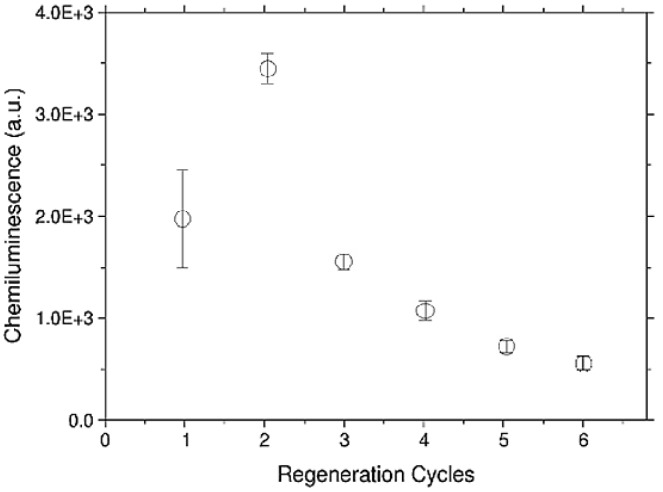
Chemiluminescence detection of AFM1 on Si_3_N_4_ substrates after regeneration cycles with 100 mM glycine-methanol solution. The data are reported as a mean value of three samples and the error bars are reported as standard deviations.

We can clearly observe a significant decrease of the sensitivity of the sensor after the first glycine-methanol injection, and again after the second one. The regeneration of the antibody-functionalized surfaces can be obtained using different approaches. These are mainly based on solutions at low pH (HCl, Glycine-HCl), or at high pH (NaOH) or at high ionic strength (MgCl2) [18]. From five up to 39 regeneration cycles are reported for the whole antibody molecule [18,19], while for Fab′ molecules, a regeneration of four cycles has been found [20]. Different solutions were tested on our Fab′-based platform. Among them, the acidic one revealed itself to be the most effective. However, acidic solutions decrease the thermal stability of Fab′ regions resulting in an unfolding of the Fab domains, and this could explain our poor regenerability. This behavior was also confirmed by experiments performed on functionalized Si_3_N_4_ flat substrate. In these, the Fab′ immobilization on silanized flat Si_3_N_4_ surfaces was performed as reported in Section 2.2. After immobilization, the surfaces were incubated with AFM1-HRP as described in Section 3.1. After the first incubation, the surfaces were washed in PBS-EDTA buffer and then regeneration with 100 mM glycine-HCl solution with 10% v/v of methanol was applied for one hour. After three washing steps in MES buffer, a new incubation with AFM1-HRP was performed. As reported in Figure 6, a decrease in the ability to again capture Aflatoxin M1 as a function of regeneration procedure was recorded. This trend is in rather good agreement with the measurements performed on functionalized aMZI (see Figure 5), considering the different regeneration procedures used for the two measurements (for aMZI, the regeneration was performed flowing the solution for 20 min, while for flat substrates an orbital shaking was applied for 1 h).

## 4. Conclusions

In this article, we designed and tested Si_3_N_4_ biosensors based on asymmetric Mach–Zehnder interferometers. We achieved a low LOD (LOD = 7 × 10^−7^ RIU) and demonstrated a high selectivity to AFM1 when Fab′ functionalization is used. The measured minimum concentration of AFM1 is still higher than the minimum concentration prescribed by regulations, which indicates the need of a preconcentration module. Other AFM1 sensors have been reported in the literature which are able to detect AFM1 concentration in the low ppt interval [21,22]. However, the sensor here proposed has the advantage of being compact, easily integrable and scalable to multi-analyte possibility.

We finally performed regeneration sensing measurements on functionalized sensors using glycine-methanol solutions and observed a clear decrease of the efficiency of the sensors after the first cleaning process. These regeneration issues were also confirmed by chemical analysis. Therefore, the proposed sensors are suitable for disposable sensors. If one would like to use them as reusable sensors, the chemistry of the regeneration should be improved with respect to the procedure here presented.

Let us clarify that the measurements we did were on purified samples. Further work still needs to be carried out if the sensor should be used with raw milk samples due to the complexity of the milk matrix. Filter and preconcentration stages should be considered to expose the sensor to a purified and concentrated sample of milk.
